# An improved novel quantum image representation and its experimental test on IBM quantum experience

**DOI:** 10.1038/s41598-021-93471-7

**Published:** 2021-07-06

**Authors:** Jie Su, Xuchao Guo, Chengqi Liu, Shuhan Lu, Lin Li

**Affiliations:** 1 College of Information and Electrical Engineering, China Agricultural University, Beijing, 100083 China; 2 Master of Health Informatics, School of Information, University of Michigan, Ann Arbor, MI 48109 USA

**Keywords:** Computational science, Computer science

## Abstract

Quantum image representation (QIR) is a necessary part of quantum image processing (QIP) and plays an important role in quantum information processing. To address the problems that NCQI cannot handle images with inconsistent horizontal and vertical position sizes and multi-channel image processing, an improved color digital image quantum representation (INCQI) model based on NCQI is proposed in this paper. The INCQI model can process color images and facilitate multi-channel quantum image transformations and transparency information processing of images using auxiliary quantum bits. In addition, the quantum image control circuit was designed based on INCQI. And quantum image preparation experiments were conducted on IBM Quantum Experience (IBMQ) to verify the feasibility and effectiveness of INCQI quantum image preparation. The prepared image information was obtained by quantum measurement in the experiment, and the visualization of quantum information was successfully realized. The research in this paper has some reference value for the research related to QIP.

## Introduction

With the development of quantum computers, quantum image processing has attracted the attention of many researchers, and more and more scholars have devoted themselves to this field of study^1–13^. Research of QIP is mainly divided into two aspects: quantum image representations and quantum image processing algorithms. Among them, the quantum image representation, as the basis of image processing, specifies the representation of images in a quantum computer. The quantum image representation model plays an important role as the basis of quantum image processing. There have been many research results on quantum image representation models, such as Qubit Lattice^14^, Entangled Image^15^, Real Ket^16^, a flexible representation for quantum images (FRQI)^17^, a novel enhanced quantum representation (NEQR)^18^, a normal arbitrary quantum superposition state (NASS)^19^, multi-channel representation of quantum image (MCRQI)^20^,quantum states for M colors and N coordinates of an image (QSMC&QSNC)^21^, simple quantum representation of infrared images (SQR)^22^, quantum log-polar images (QUALPI)^23^, Caraiman’s quantum Image representation (CQIR)^24^, multi-channel quantum images (MCQI)^25^, Improved NEQR (INEQR)^26^, a generalized model of NEQR (GNEQR)^27^, a novel quantum representation of color digital images (NCQI)^28^, a bitplane representation of quantum images (BRQI)^29^, a new quantum representation model of color digital images (QRCI)^30^, a quantum representation model for multiple images (QRMMI)^31^, quantum representation of multi wavelength images (QRMW)^32^, an optimized quantum representation for color digital images (OCQR)^33^, an improved FRQI model (FRQCI)^34^, a digital RGB multi-channel representation for quantum colored images (QMCR)^35^, an improved flexible representation of quantum images (IFRQI)^36^, a quantum block image representation (QBIR)^37^, order-encoded quantum image model (OQIM)^38^, quantum indexed image representation (QIIR)^39^, and a double quantum color images representation model (DRQCI)^40^ and so on. These quantum image representations encode color pixels as well as positions in different ways, making them somewhat different in terms of image processing applications and algorithmic complexity. However, in terms of storage methods and resource requirements, these quantum image representations basically use the same strategy of using energy converters, similar to function mapping, to convert energy values into a collection of quantum states. If the energy is an electromagnetic wave that can be detected and recorded, then the frequency can be converted into a quantum state representing color information; if the energy is infrared radiation, then the conversion of the radiant energy of the object to a quantum state storing color information can be achieved by measuring the intensity of the radiation and thus. Different quantum image representations enable the capture, processing and acquisition of images in different formats in quantum systems for various practical applications. There are some differences in image color information types and position information types processed by different quantum image representations, as well as in the compilation mode of image color information taken. Therefore, quantum image representations can be divided according to image color model, image coordinate model and image color information compilation model^41^. If the image is divided according to the color model, it can be divided into quantum image representations based on binary information, quantum image representations based on gray level, quantum image representations based on RGB model and quantum image representations based on infrared image. Among them, the structure of quantum image representations based on binary information is very simple, easy to store, acquire and other related operations, and it is mainly applied to image segmentation, binarization and other operations. Quantum image representations based on gray levels represent somewhat more color information than quantum image representations based on binary information, such as the FRQI. Quantum image representations based on the RGB model are capable of representing color images, which can be divided into two categories, one of which uses two sets of quantum ground states to represent color information and position information, such as the QSMC&QSNC, and the other uses angles to represent RGB information and represents the image by performing tensor product operation with position information, such as the MCQI. Quantum image representations based on infrared images are different from other representations which are based on encoding infrared images, such as SQR. In addition, they can be classified according to the image coordinate system into quantum image representations based on Cartesian coordinate system, quantum image representations based on logarithmic polar coordinate system, and multidimensional quantum image representations. Both the Qubit Lattice and the Real Ket are quantum image representations based on a two-dimensional Cartesian coordinate system, which are intuitive in description and convenient for image transformation operations. For example, the FRQI is based on a two-dimensional Cartesian coordinate system, and it can implement a series of geometric transformations such as two-point transformation, image flip, and orthogonal transformation. In addition, quantum image representations based on logarithmic polar coordinate system are convenient for complex affine transformations such as image rotation and blooming. For example, the QUALPI can store and process images in logarithmic polar coordinates, and easily implement central symmetry, axisymmetric and rotational transformations, etc. Multidimensional quantum image representations can realize image storage in multidimensional Cartesian coordinate system, such as the NAQSS, which can represent multidimensional color images. If the image is divided according to the image color information compilation model, the quantum image representations can be divided into two categories: one is to use the Angle coefficient of quantum bits to compile the color information; the other is to use the ground state of quantum sequence to compile the color information. The Qubit Lattice, the FRQI, and the QSMC&QSNC belong to the former category. They have the same compiling method for color information, but different compiling methods for location information. The Qubit Lattice does not specify the exact way to compile the position information; the FQRI stores the position information of each pixel point by a 2n-dimensional quantum sequence of basis; the QSMC & QSNC store the position information in the angular coefficients of quantum bits by a bijection function. After these quantum image representations, many new types of quantum image representations emerged which use the ground state of quantum sequences to compile color information, such as the CQIR and the NEQR. In this paper, a new improved quantum expression INCQI for color digital images is proposed, which improves on the NCQI and adds auxiliary quantum bits to facilitate image processing operations ^28^. In addition, the quantum representation uses ${\text{n}}_{1}  + {\text{n}}_{2}  + 4q$ qubits to achieve the purpose of compiling multi-channel color information, and it has not only the geometric operations (such as two-point transformation, image flip and rotation, etc.) that the FRQI have, but also those functions of the MCQI for multi-channel color operations, and it can use NOT gates, CNOT gates, and SWAP gates to implement the four channels of R, G, B, and $\alpha$ operations. This expression can solve the problem that the scaled image size is no longer in the form of $2^{n}  \times 2^{n}$ when the horizontal scaling of the image and the vertical blooming scale are not equal, and it can handle well for quantum images of the form similar to $2^{{n_{1} }}  \times 2^{{n_{2} }}$. Besides, the INCQI-based model can easily perform more image operations, especially some complex color transformations, such as addition (subtraction), compression, complementary operations, feature extraction, etc. Due to these advantages, the INCQI is more flexible and more suitable for quantum image processing operations.

The remaining part of the paper proceeds as follows. “Models” introduces the relevant quantum image representation model NCQI and the INCQI. “Experiment” describes the INCQI for simulation experiments on the IBM quantum experience. "Discussion" is the discussion.  "Conclusion" is the conclusions.

## Models

In this paper, the INCQI is proposed by improving on the NCQI. In this section, the reference quantum image representation NCQI and the improved quantum image representation INCQI are described in detail.

### The referenced quantum image representation: NCQI

Assuming that the range of location information $x$, $y$ are $[0,2^{n}  - 1]$ , the NCQI is defined as

1 $$ |I\rangle  = \frac{1}{{2^{n} }}\sum\limits_{{y = 0}}^{{2^{n}  - 1}} {\sum\limits_{{x = 0}}^{{2^{n}  - 1}} | } c(y,x)\rangle  \otimes |yx\rangle ,$$

where $c(y,x)$ represents the color value of the corresponding pixel, which has the binary form $R_{{q - 1}}  \cdots R_{0} G_{{q - 1}}  \cdots G_{0} B_{{q - 1}}  \cdots B_{0}$ , as shown below.

2 $$ |c(y,x)\rangle  = |\underbrace {{R_{{q - 1}}  \ldots R_{0} }}_{{{\text{Red}}}}\underbrace {{G_{{q - 1}}  \cdots G_{0} }}_{{{\text{Green}}}}\underbrace {{B_{{q - 1}}  \cdots B_{0} }}_{{Blue}}\rangle. $$

The range of values for each channel in $(R,G,B)$ is $\left[ {0,2^{q}  - 1} \right]$. $x$ is the horizontal position, $y$ is the vertical position, and $c(y,x)$ is the color information. The tensor multiplication of these three partial quantum sequences forms the ground state of the NCQI. Using the NCQI to store a color image of size $2^{n}  \times 2^{n}$ requires $2n + 3q$ quantum bits.

In the NCQI, the horizontal position range and the vertical position range in the position information are equal, so there is a limitation on the image's size ratio to be processed, and it is not possible to process images with an aspect ratio other than 1. In addition, the NCQI contains quantum bits for R, G, B channels, which are convenient for processing color images, but no auxiliary quantum bits are reserved for image processing operations, which are not convenient for image transformation operations. Therefore, in this paper, these problems of the NCQI are improved and can be handled well for quantum images of this form of size $2^{{n_{1} }}  \times 2^{{n_{2} }}$.

### The improved quantum image expression: INCQI

In response to the problem that the NCQI cannot handle images with inconsistent horizontal and vertical position sizes and subsequent transformation operations of quantum images, we improved the NCQI and proposed the INCQI. The INCQI is broadly similar to the NCQI, in which the range of values for horizontal and vertical positions is more flexible, without the 1:1 restriction, but with an arbitrary $n_{1} :n_{2}$ ratio. The INCQI is defined as

3 $$ |I\rangle  = \frac{1}{{2^{{\frac{{n_{1}  + n_{2} }}{2}}} }}\sum\limits_{{y = 0}}^{{2^{{n_{1} }}  - 1}} {\sum\limits_{{x = 0}}^{{2^{{n_{2} }}  - 1}} | } c(y,x)\rangle  \otimes |yx\rangle ,$$

where

4 $$ |c(y,x)\rangle  = |\underbrace {{R_{{q - 1}}  \ldots R_{0} }}_{{{\text{Red}}}}\underbrace {{G_{{q - 1}}  \cdots G_{0} }}_{{{\text{Green}}}}\underbrace {{B_{{q - 1}}  \cdots B_{0} }}_{{Blue}}\underbrace {{\alpha _{{q - 1}}  \cdots \alpha _{0} }}_{\alpha }\rangle, $$

**Figure 1:**
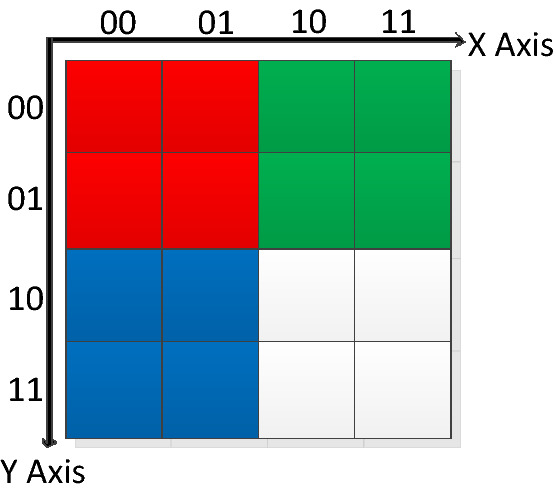
A color image of 4 × 4 size.

$c(y,x)$ indicates the pixel value at position $(y,x)$, $x$ is the horizontal position, and $y$ is the vertical position. Also, $\alpha$ is an accessory quantum bit to store the intermediate value of each pixel after transformation, such as the threshold value in image segmentation. Compared to the NCQI, the INCQI contains four channels: red, green, and blue color channels, and the remaining one is $\alpha$ calculation channel, which can save pixel calculation results. The range of all 4 channels is $\left[ {0,2^{q}  - 1} \right]$, e.g. for images with pixels in the range 0–255, $q = 8$. Besides, $|yx\rangle$ is expanded in binary sequence in the form shown as

5 $$ |YX\rangle  = |Y\rangle |X\rangle  = |y_{0} y_{1}  \ldots y_{{n_{1}  - 1}} \rangle |x_{0} x_{1}  \ldots x_{{n_{2}  - 1}} \rangle, $$

where $y_{i} ,x_{i}  \in \{ 0,1\}$ , $Y$ and $X$ denote the $Y$-axis and $X$-axis, respectively. For a color picture (see Fig. 1) of size ${\text{4}} \times {\text{4}}$, use the above INCQI to express it as shown in Eq. (6) below.

6 $$ \begin{gathered}   |I\rangle {\text{ = }}\frac{1}{{\sqrt {2^{{{\text{2 + 2}}}} } }}\left[ {\left| {\underbrace {{111111111}}_{R}\underbrace {{000000000}}_{G}\underbrace {{00000000}}_{{\text{B}}}\underbrace {{00000000}}_{\alpha }} \right\rangle  \otimes ((0000) + |0001\rangle  + |0100\rangle  + |0101\rangle )} \right. \hfill \\   \begin{array}{*{20}c}    {\begin{array}{*{20}c}    {} & {}  \\   \end{array} } & {} & {\text{ + }}  \\   \end{array} \left[ {\left| {\underbrace {{00000000}}_{R}\underbrace {{111111111}}_{G}\underbrace {{00000000}}_{{\text{B}}}\underbrace {{00000000}}_{\alpha }} \right\rangle  \otimes ((00{\text{1}}0) + |00{\text{1}}1\rangle  + |01{\text{1}}0\rangle  + |01{\text{1}}1\rangle )} \right. \hfill \\   \begin{array}{*{20}c}    {\begin{array}{*{20}c}    {} & {}  \\   \end{array} } & {} & {\text{ + }}  \\   \end{array} \left[ {\left| {\underbrace {{00000000}}_{R}\underbrace {{00000000}}_{G}\underbrace {{111111111}}_{{\text{B}}}\underbrace {{00000000}}_{\alpha }} \right\rangle  \otimes ((10{\text{0}}0) + |10{\text{0}}1\rangle  + |11{\text{0}}0\rangle  + |11{\text{0}}1\rangle )} \right. \hfill \\   \begin{array}{*{20}c}    {\begin{array}{*{20}c}    {} & {}  \\   \end{array} } & {} & {\text{ + }}  \\   \end{array} \left[ {\left| {\underbrace {{111111111}}_{R}\underbrace {{111111111}}_{G}\underbrace {{111111111}}_{{\text{B}}}\underbrace {{00000000}}_{\alpha }} \right\rangle  \otimes ((10{\text{1}}0) + |10{\text{1}}1\rangle  + |11{\text{1}}0\rangle  + |11{\text{1}}1\rangle )} \right. \hfill \\  \end{gathered} .$$

(1) The process of INCQI quantum image preparation

**Figure 2:**
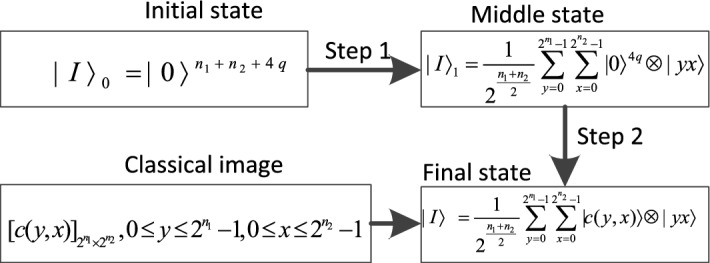
Flow chart of the INCQI quantum image preparation.

From the definition of the INCQI, it takes $n_{1}  + n_{2}  + 4q$ quantum bits to store a color image of size $2^{{n_{1} }}  \times 2^{{n_{2} }}$ using the INCQI. Two steps are required to prepare an INCQI image, and the flowchart is shown in Fig. 2.

In Step 1, the operation operator ${\text{U}}_{1}$ is constructed by tensor operations on two common single quantum gates $I$ and $H$, which are represented as follows.

7 $$ I = \left[ {\begin{array}{*{20}l}    1 \hfill & 0 \hfill  \\    0 \hfill & 1 \hfill  \\   \end{array} } \right], $$

8 $$ H = \frac{1}{{\sqrt 2 }}\left[ {\begin{array}{*{20}c}    1 & 1  \\    1 & { - 1}  \\   \end{array} } \right] ,$$

9 $$ U_{1}  = I^{{ \otimes 4q}}  \otimes H^{{ \otimes n_{1}  + n_{2} }} .$$

Then use the operator ${\text{U}}_{1}$ to act on $|I\rangle _{0}$ as follows.

10 $$ \begin{gathered}   U_{1} \left( {|I\rangle _{0} } \right) = I^{{ \otimes 4q}}  \otimes H^{{ \otimes n_{1}  + n_{2} }} \left( {|0\rangle ^{{n_{1}  + n_{2}  + 4q}} } \right) \hfill \\   {\text{             }} = |0\rangle ^{{4q}}  \otimes \frac{1}{{\sqrt {2^{{n_{1} }} } }}\sum\limits_{{y = 0}}^{{2^{{n_{1} }}  - 1}} | y\rangle  \otimes \frac{1}{{\sqrt {2^{{n_{2} }} } }}\sum\limits_{{x = 0}}^{{2^{{n_{2} }}  - 1}} | x\rangle  \hfill \\   {\text{               = }}\frac{1}{{2^{{\frac{{n_{1}  + n_{2} }}{2}}} }}\sum\limits_{{y = 0}}^{{2^{{n_{1} }}  - 1}} {\sum\limits_{{x = 0}}^{{2^{{n_{2} }}  - 1}} | } 0\rangle ^{{4q}}  \otimes |yx\rangle  = |I\rangle _{1}  \hfill \\  \end{gathered} .$$

The intermediate state $|I\rangle _{1}$ thus obtained is an empty quantum superposition state.

In Step2, $|I\rangle _{1}$ is assigned a value, and since the image size is $2^{{n_{1} }}  \times 2^{{n_{2} }}$ , $n_{1}  + n_{2}$ suboperations are performed to set each pixel. The pixel at the first $(y,x)$ position can be set by $\Omega _{{yx}}$.

11 $$ \Omega _{{yx}}  =  \otimes _{{i = 0}}^{{4q - 1}} \Omega _{{yx}}^{i}. $$

In Eq. (11), $\Omega _{{yx}}^{i} :|0\rangle  \to \left| {0 \oplus C_{i} } \right\rangle$, the quantum bits of each color value in this operation are processed according to the binary in color $c(y,x)$. When ${\text{C}}_{i} {\text{ = 1}}$, the $i$th quantum bit will be operated by $(n_{1}  + n_{2} ) - {\text{CNOT}}$ quantum control gate, otherwise no operation will be performed. Besides,

12 $$ \Omega _{{yx}} :\mathop {\mathop  \otimes \limits^{{4q - 1}} }\limits_{{i = 0}} |0\rangle  \to \mathop {\mathop  \otimes \limits^{{4q - 1}} }\limits_{{i = 0}} \left| {0 \oplus C_{i} } \right\rangle  = \mathop {\mathop  \otimes \limits^{{4q - 1}} }\limits_{{i = 0}} \left| {C_{i} } \right\rangle  = |c(y,x)\rangle, $$

where $\left| {C_{i} } \right\rangle  = \left| {R_{i} } \right\rangle ,i = 3q, \ldots ,4q - 1$, $\left| {C_{i} } \right\rangle  = \left| {G_{i} } \right\rangle ,i = 2q, \ldots ,3q - 1$,$\left| {C_{i} } \right\rangle  = \left| {B_{i} } \right\rangle ,i = q, \ldots ,2q - 1$,and $\left| {C_{i} } \right\rangle  = \left| {\alpha _{i} } \right\rangle ,i = 0, \ldots ,q - 1$. For each suboperation in Step 2, the unitary operation operator ${\text{U}}_{{yx}}$ can be represented as

13 $$ U_{{yx}}  = \left( {I^{{ \otimes 4q}}  \otimes \sum\limits_{{j = 0}}^{{2^{{n_{{_{1} }} }}  - 1}} {\sum\limits_{{i = 0,ji \ne yx}}^{{2^{{n_{{_{2} }} }}  - 1}} | } ji\rangle \langle ji|} \right) + \Omega _{{yx}}  \otimes |yx\rangle \langle yx| $$

Use the operator ${\text{U}}_{{yx}}$ to act on $|I\rangle _{1}$ as follows.

14 $$ \begin{gathered}   U_{{yx}} \left( {|I\rangle _{1} } \right) = \left[ {\left( {I^{{ \otimes 4q}}  \otimes \sum\limits_{{j = 0}}^{{2^{{n_{{_{1} }} }}  - 1}} {\sum\limits_{{i = 0,ji \ne yx}}^{{2^{{n_{{_{2} }} }}  - 1}} | } ji\rangle \langle ji|} \right) + \Omega _{{yx}}  \otimes |yx\rangle \langle yx|} \right] \hfill \\   {\text{                  }} \times \left( {\frac{1}{{2^{{\frac{{n_{1}  + n_{2} }}{2}}} }}\sum\limits_{{y = 0}}^{{2^{{n_{1} }}  - 1}} {\sum\limits_{{x = 0}}^{{2^{{n_{2} }}  - 1}} | } 0\rangle ^{{ \otimes {\text{4}}q}} |yx\rangle } \right) \hfill \\   {\text{               }} = \frac{1}{{2^{{\frac{{n_{1}  + n_{2} }}{2}}} }}\sum\limits_{{j = 0}}^{{2^{{n_{1} }}  - 1}} {\sum\limits_{{i = 0,ji \ne yx}}^{{2^{{n_{2} }}  - 1}} | } 0\rangle ^{{ \otimes 4q}} |ji\rangle  + \Omega _{{yx}} |0\rangle ^{{ \otimes 4q}} |yx\rangle  \hfill \\   {\text{                }} = \frac{1}{{2^{{\frac{{n_{1}  + n_{2} }}{2}}} }}\sum\limits_{{j = 0}}^{{2^{{n_{1} }}  - 1}} {\sum\limits_{{i = 0,ji \ne yx}}^{{2^{{n_{2} }}  - 1}} | } 0\rangle ^{{ \otimes 4q}} |ji\rangle  + |c(y,x)\rangle |yx\rangle  \hfill \\  \end{gathered} .$$

The above operation can only set the relevant pixel values. In order to set the color values of all pixels, ${\text{U}}_{{\text{2}}}$ is introduced, whose definition is as

15 $$ U_{2}  = \prod\limits_{{y = 0}}^{{2^{{n_{1} }}  - 1}} {\prod\limits_{{x = 0}}^{{2^{{n_{2} }}  - 1}} {U_{{yx}} } } .$$

By Step 1 and Step 2 above, an INCQI quantum image is prepared.

(2) Time complexity validation of the preparation process

To verify the correctness of the INCQI, we compile a color image of size $2^{{n_{1} }}  \times 2^{{n_{2} }}$ with a pixel range between $\left[ {0,2^{q}  - 1} \right]$ into an INCQI quantum image, preparing a total consumption of no more than $O\left( {4q + n_{1}  + n_{2}  + 4q(n_{1}  + n_{2} ) \cdot 2^{{n_{1}  + n_{2} }} } \right)$. The proof is shown below.

#### Proof

Since the total preparation process includes Step 1 and Step 2, the time complexity analysis mainly includes: First, in Step 1, since $4q + n_{1}  + n_{2}$ single quantum gate is included, the cost of operator operation ${\text{U}}_{1}$ is $O\left( {4q + n_{1}  + n_{2} } \right)$. Secondly in Step 2, the operator ${\text{U}}_{2}$ contains $2^{{n_{1}  + n_{2} }}$ sub-operations ${\text{U}}_{{yx}}$, each ${\text{U}}_{{yx}}$ performs a quantum operation $\Omega _{{yx}}$ , the color value $c(y,x)$ in which if ${\text{C}}_{i}  = 1$, where $i\,\,=\,\,0, \ldots 4q\,-\,1$, then the $i$-th bit in the quantum sequence $\mathop {\mathop  \otimes \limits^{{4q - 1}} }\limits_{{i = 0}} \left| {C_{i} } \right\rangle$ is applied to a control quantum gate $(n_{1}  + n_{2} ) - {\text{CNOT}}$. The control quantum gate can be decomposed into single quantum gates with no more than $O(n_{1}  + n_{2} )$. Therefore, the total time complexity of ${\text{U}}_{{yx}}$ is $O\left( {4q(n_{1}  + n_{2} )} \right)$, and since there are $2^{{n_{1}  + n_{2} }}$ ${\text{U}}_{{yx}}$ such sub-operations in it, the time spent in Step 2 does not exceed $O\left( {4q(n_{1}  + n_{2} ) \cdot 2^{{n_{1}  + n_{2} }} } \right)$. Combining the above Step 1 and Step 2 time consumption analysis, the total consumption of preparing such an INCQI quantum image is no more than $O\left( {4q + n_{1}  + n_{2}  + 4q(n_{1}  + n_{2} ) \cdot 2^{{n_{1}  + n_{2} }} } \right)$.

The INCQI proposed in this paper has similar time complexity in preparation as the NCQI. A factor of about 2 reduces the preparation time complexity of INCQI quantum images compared to MCRQI. Therefore, the construction of the INCQI is verified to be reasonable from the theoretical proof.

(3) Channel operation of The INCQI

The INCQI has four channels: R, G, B and $\alpha$. The first three channels support color image processing. The auxiliary bits used by the latter channel $\alpha$ can facilitate more image processing operations and channel switching operations, and can also represent the transparency of the image. The auxiliary quantum bits can represent the color transparency information of the image and support the 4-channel representation of the image. In addition, the auxiliary quantum bits can be used to save the calculation results of RGB color channels, so that the information of the original channels can be well preserved when channel exchange and channel calculation are performed, avoiding the loss of the original channel information of the image after channel calculation and facilitating the detailed operation of the image. The $\alpha$ channel can record the selection area by transparency representation, such as recording the segmentation area of the image. The content of the Alpha channel represents not the color of the image, but the selection area, where the white color indicates the completely selected area, and the black color is the non-selected area, and different levels of gray scale represent different selection percentages, and there can be up to 256 levels of gray scale. Some of the channel operations are defined below.

#### Definition 1

The Channel operation (CO) on INCQI are the operations $CO_{R}$, $CO_{G}$, $CO_{B}$, $CO_{\alpha }$(changing the value of R-Channel, G-Channel, B-Channel, and $\alpha$-Channel respectively) which when applying on $|I\rangle$ produce the output of the following form

16 $$ \begin{gathered}   CO_{Z} (|I\rangle )\,\,=\,\,|I{\prime }\rangle\,=\,\frac{1}{{2^{{\frac{{n_{1}  + n_{2} }}{2}}} }}\sum\limits_{{y = 0}}^{{2^{{n_{1} }}  - 1}} {\sum\limits_{{x = 0}}^{{2^{{n_{2} }}  - 1}} {CO_{Z} (|} } c(y,x)\rangle ) \otimes |yx\rangle  \hfill \\    = \frac{1}{{2^{{\frac{{n_{1}  + n_{2} }}{2}}} }}\sum\limits_{{y = 0}}^{{2^{{n_{1} }}  - 1}} {\sum\limits_{{x = 0}}^{{2^{{n_{2} }}  - 1}} {CO_{Z} (|} } \underbrace {{R_{{q - 1}}  \ldots R_{0} }}_{{{\text{Red}}}}\underbrace {{G_{{q - 1}}  \cdots G_{0} }}_{{{\text{Green}}}}\underbrace {{B_{{q - 1}}  \cdots B_{0} }}_{{Blue}}\underbrace {{\alpha _{{q - 1}}  \cdots \alpha _{0} }}_{\alpha }\rangle ) \otimes |yx\rangle  \hfill \\  \end{gathered} $$

where $$Z \in \{ R,G,B\}$$, and

17 $$\begin{gathered}   CO_{R} (\left| {c(y,x)} \right\rangle )\,=\,|\underbrace {{R^{\prime}_{{q - 1}}  \ldots R^{\prime}_{0} }}_{{{\text{Red}}}}\underbrace {{G_{{q - 1}}  \cdots G_{0} }}_{{{\text{Green}}}}\underbrace {{B_{{q - 1}}  \cdots B_{0} }}_{{Blue}}\underbrace {{\alpha _{{q - 1}}  \cdots \alpha _{0} }}_{\alpha }\rangle ; \hfill \\   CO_{G} (\left| {c(y,x)} \right\rangle )\,=\,|\underbrace {{R_{{q - 1}}  \ldots R_{0} }}_{{{\text{Red}}}}\underbrace {{G^{\prime}_{{q - 1}}  \cdots G^{\prime}_{0} }}_{{{\text{Green}}}}\underbrace {{B_{{q - 1}}  \cdots B_{0} }}_{{Blue}}\underbrace {{\alpha _{{q - 1}}  \cdots \alpha _{0} }}_{\alpha }\rangle ; \hfill \\   CO_{B} (\left| {c(y,x)} \right\rangle )\,=\,|\underbrace {{R_{{q - 1}}  \ldots R_{0} }}_{{{\text{Red}}}}\underbrace {{G_{{q - 1}}  \cdots G_{0} }}_{{{\text{Green}}}}\underbrace {{B^{\prime}_{{q - 1}}  \cdots B^{\prime}_{0} }}_{{Blue}}\underbrace {{\alpha _{{q - 1}}  \cdots \alpha _{0} }}_{\alpha }\rangle ; \hfill \\   CO_{\alpha } (\left| {c(y,x)} \right\rangle )\,=\,|\underbrace {{R_{{q - 1}}  \ldots R_{0} }}_{{{\text{Red}}}}\underbrace {{G_{{q - 1}}  \cdots G_{0} }}_{{{\text{Green}}}}\underbrace {{B_{{q - 1}}  \cdots B_{0} }}_{{Blue}}\underbrace {{\alpha ^{\prime}_{{q - 1}}  \cdots \alpha ^{\prime}_{0} }}_{\alpha }\rangle  \hfill \\  \end{gathered} .$$

Obviously, we can use the combination of NOT gate to implement the above channel operation.

## Experiment

In this section, we use IBMQ (IBM Quantum Experience) for the experimental implementation of the INCQI quantum image preparation, measurement, and imaging of quantum sequences.

### The introduction of IBMQ

IBMQ is a quantum computing cloud platform opened by IBM in 2017, which contains the quantum information software toolkit Qiskit^42^. Researchers can use Qiskit to create quantum computing programs, compile them, and execute them on a real quantum simulator on IBMQ and a local virtual quantum simulator. On IBMQ, users can compile the Python language into OpenQASM language (it is an intermediate language proposed by IBM for quantum experiments, similar to assembly language in classical computers) according to the environment management provided by Qiskit and Anaconda. Then the quantum circuits are generated and various operations of the quantum circuits are executed on the quantum computer, quantum measurements are performed, and finally the measurement results are output.

Currently, there are nine quantum simulators on IBMQ, and these machines that can run quantum algorithms are usually referred to as backend. Currently, IBMQ supports a maximum of 8192 executions of quantum lines, and the backend "ibmq_qasm_simulator" is limited to 10,000 s (about 2.5 h) for job submissions. Table 1 shows the names of the five quantum simulators, the number of quantum bits allowed and the information about the population used. For the specific simulations, we use the virtual quantum simulator on the local computer and run the same results as the quantum computing results on the cloud platform.

**Table 1:** Quantum simulator on IBMQ.

Quantum simulator	Quantum bits	Population of users
ibmq_santiago	5	All users
ibmq_athens	5	All users
ibmq_vigo	5	All users
ibmq_valencia	5	All users
ibmq_16_melbourne	15	All users
ibmq_ourense	5	All users
ibmqx2	5	All users
ibmq_armonk	1	All users
ibmq_qasm_simulator	32	All users

On the local backend, the configuration of the PC is shown in Table 2. When using the local backend in Qiskit, it is mainly called by the statement "backend = provider.get_backend('local_qasm_simulator')".

**Table 2:** Hardware simulation environment.

Parameters of the category	The parameter value
CPU	Intel(R) Core(TM) i5-8265U CPU @ 1.60 GHz 1.80 Ghz
Memory	16 GB
The operating system	Windows 10 64bits

Due to the physical noise, quantum bits are prone to errors in the actual operation of quantum computers. To solve the noise problem in quantum computer, extra qubits are provided for each qubit as a backup. To simulate a quantum system experimentally on a classical computer, each practical qubit might require 1,000 or more backup qubits. Table 3 provides the quantum programming language for IBM Quantum Lab to correspond to the memory sizes required for different quantum bits.

**Table 3:** Memory sizes corresponding to different sizes of quantum bits for classical computer simulations.

Qubits	Memory/GB	Qubits	Memory/GB
25	0.54	31	34.36
26	1.07	32	68.72
27	2.15	33	137
28	4.29	34	275
29	8.59	35	550
30	17.18	36	1100

In the experiment, this paper implements INCQI-based quantum image preparation and processing through the IBMQ platform and quantum programming language. The whole process mainly completes the transformation process from classical image information to superposition state of quantum image information and the probability amplitude results of the superposition state are obtained by measurement. The experiments in this paper mainly run the quantum image algorithm in the background of "ibmq_qasm_simulator".

### Design of quantum circuit for the INCQI image preparation

This section will introduce the quantum circuit of the INCQI designed based on quantum gates in Qiskit and quantum computing tools.

**Figure 3:**
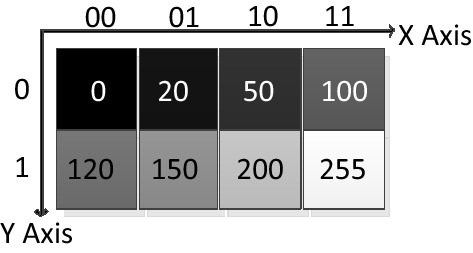
A gray image of 2 × 4 size.

The analysis of the memory size required to simulate quantum algorithms in the above section shows that for each additional quantum bit in the quantum circuit, the space of memory needed increases nearly doubled, which will make our simulation more difficult. In addition, since the IBMQ platform supports quantum operations with a maximum of 32 quantum bits, this paper will prepare and design quantum circuits for INCQI images with a 2 × 4 size grayscale image (see Fig. 3) as an example.

**Figure 4:**
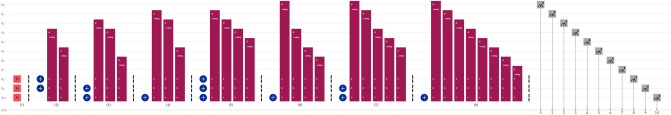
Quantum circuit of 2 × 4 size gray image preparation based on the INICQI.

For a grayscale image of size 2 × 4, the number of quantum bits required for its direction and orientation are 2 and 1, respectively, and 8 quantum bits are needed to represent the grayscale information. For a color image, 3 × 8 = 24 quantum bits are required to represent the color information. Also, if the $\alpha$ computational channel is added, 4 × 8 = 32 quantum bits are needed to represent the color information and computational information. According to the method and steps for the preparation of INCQI quantum images described in the previous sections of this paper, firstly, we need to apply the H-gate transformation to the three quantum bits representing the position information, so that the 8-state superposition state system can be obtained. Then, the sequence of initial states representing the grayscale information in the quantum image is converted to the quantum state of the pixel information of the actual image according to the mapping relationship between the coordinates and the grayscale information. For example, the grayscale information at $(Y,X)$ coordinates $(01,00)$ is converted from $\left| 0 \right\rangle$ to $\left| {120} \right\rangle$. After all the grayscale information corresponding to each position is converted by quantum gates, the classical image is converted into an INCQI quantum image for storage and representation. The quantum circuit representation of the above 2 × 4 size grayscale image preparation is shown in Fig. 4.

**Figure 5:**
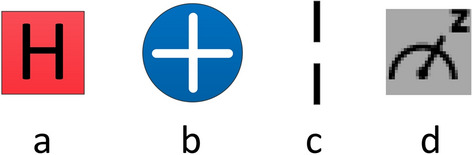
The icon description in the IBMQ quantum circuit. **(a)** The Hadamard gate, **(b)** not gate, **(c)** barrier, using it prevents the transmission from crossing the line, which does not affect the theoretical results of the circuit, but may affect the runtime or accuracy, this is because the function of the barrier is to prevent any back-end tool from optimizing the circuit on that barrier, here it is used only to split each processing module. **(d)** Measuring operation.

In Fig. 4, q0—q7 are the color information quantum bits, which represent the color information from high to low in order; q_8_ represents the position information $y$; q_9_ and q_10_ represent the high and low bits of the position information $x$, respectively. The descriptions about the different icons in the figure are shown in Fig. 5.

**Figure 6:**
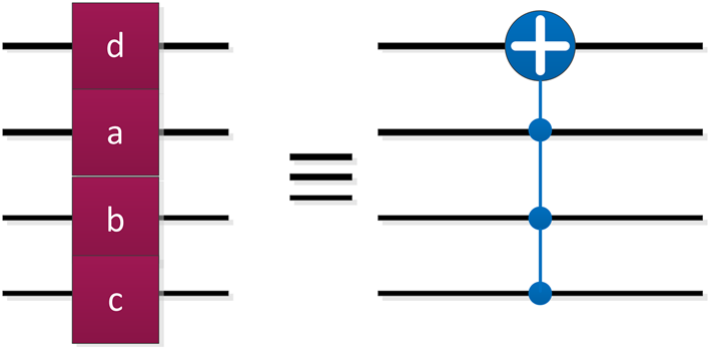
The "coding" quantum control gate.

Besides, the quantum gates represented by "coding" in the circuit are shown in Fig. 6, where a,b,c represent the control quantum bits and d is the target bit. In fact, "coding" stands for 4-qubits CNOT.

In the above 2 × 4 size grayscale image, there are a total of 8 pixels to be encoded, and the corresponding binary sequence of grayscale information is represented as Table 4.

**Table 4:** 2 × 4 size gray image color information encoding.

YX_H_X_L_	000	001	010	011	100	101	110	111
F(YX_H_X_L_)	00000000	00010100	00110010	01100100	01111000	10,010,110	11,001,000	11,111,111

In the quantum preparation circuit realized above, a lot of 3-CNOT gates are used. It is not a basic quantum gate, in which three quantum bits are used as the control bit and one is used as the target bit. If the size of the image is $2^{{n_{1} }}  \times 2^{{n_{2} }}$, where $n_{1} ,n_{2}  \ge 2$, then during the preparation of the quantum image of the INCQI, multiple multi-bit CNOT gates, namely $(n_{1}  + n_{2} )$-CNOT gates, will appear in the quantum circuit. Yang et al. proposed that each $k$-CNOT gate can be decomposed into $4k - 8$ Toffoli gates, and $k - 2$ auxiliary quantum bits are also required. Then for preparing an image of size $2^{{n_{1} }}  \times 2^{{n_{2} }}$ and grayscale range $[0,2^{q}  - 1]$, considering the highest complexity, $q$ $(n_{1}  + n_{2} )$-CNOT gates per pixel are needed to achieve this, and since each $(n_{1}  + n_{2} )$-CNOT gate can be decomposed into $4(n_{1}  + n_{2} ) - 8$ Toffoli gates, the upper limit of base gates needed for the whole process is $2^{{n_{1} }}  \times 2^{{n_{2} }}  \times q \times [4(n_{1}  + n_{2} ) - 8]$. Besides, for the problem of the large increase in the number of auxiliary quantum bits required as the image size increases, the quantum circuit can be optimized by using a zero-setting gate ^18^, so that only two auxiliary quantum bits are always required, regardless of the increase in image size.

### The experimental test on IBM quantum experience

After designing the quantum circuit for preparing the INCQI image for the above 2 × 4 size grayscale image, the next test was performed in the Qiskit simulation system of IBM Quantum Experience. When the quantum circuit was run in Qiskit, the output quantum sequence from left to right corresponds to the quantum circuit from bottom to top. The image of this processing is shown below in matrix form.

18 $$ f_{{2 \times 4}}  = \left[ {\begin{array}{*{20}c}    0 & {20} & {50} & {100}  \\    {120} & {150} & {200} & {255}  \\   \end{array} } \right] $$

**Figure 7:**
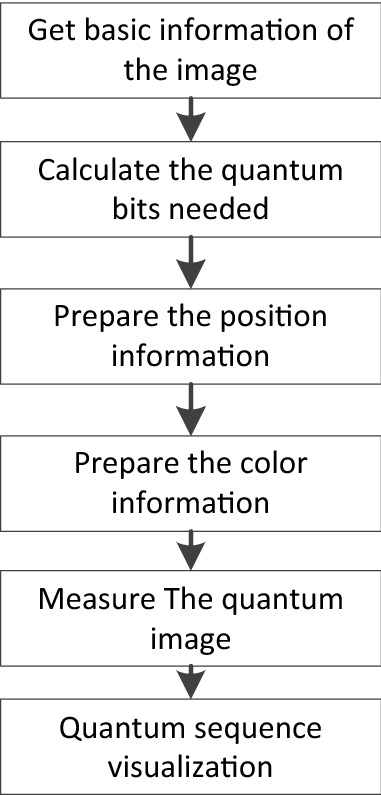
Flow chart of the experiment.

The overall flow of this experiment is shown in Fig. 7.

The whole experimental process is mainly to verify the INCQI. In the quantum computer environment, the feasibility of the quantum image preparation process of the INCQI is verified, and the prepared image information is obtained through quantum measurement operation to realize the process of quantum information visualization and fully demonstrate the advantages of quantum computation.

(1) Get basic information of the image

In this step, we mainly analyze the processed grayscale image to obtain its basic information. Define the image to be processed as F to facilitate subsequent representation. F has a total of 8 grayscale pixels, each of which ranges from [0,255]. Then, the gray level information of F is converted into a binary digit size of 8, the width information n1 is 1, and the length information n2 is 2.

(2) Calculate the quantum bits needed

The corresponding quantum bits are set according to the basic information of the image obtained in the previous step. Grayscale information requires 8 qubits, and position information requires 3 qubits.

(3) Prepare the position information

The initial state of the quantum bit of known position information is $\left| {\text{0}} \right\rangle$, and H gate transformation is performed on the position quantum bit to prepare position information according to the preparation process of the quantum image of the INCQI. The H gate transformation is carried out for the three quantum bits in the position information of F, and the superposition state of the eight components is obtained, which is expressed as follows.

19 $$ \left| Q \right\rangle  = \left[ {\begin{array}{*{20}c}    {\left| {00000000000} \right\rangle } & {\left| {00100000000} \right\rangle } & {\left| {01000000000} \right\rangle } & {\left| {01100000000} \right\rangle }  \\    {\left| {10000000000} \right\rangle } & {\left| {10100000000} \right\rangle } & {\left| {11000000000} \right\rangle } & {\left| {11100000000} \right\rangle }  \\   \end{array} } \right] $$

Since the Qiskit quantum system in IBMQ runs the quantum circuit with the output quantum sequence from left to right corresponding to the quantum circuit from bottom to top, the first 3 bits of each quantum bit in Eq. (19) represent the position information and the last 8 bits represent the grayscale information. Besides, the position information is obtained in this step using only the H-gate, while the grayscale information remains zero.

(4) Prepare the color information

**Figure 8:**
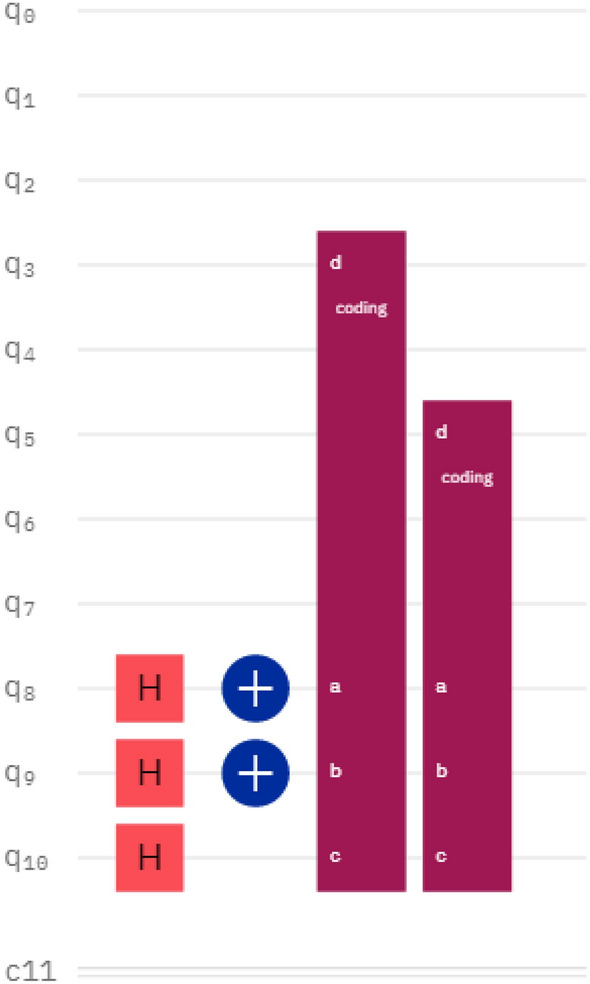
The quantum circuit prepared with the gray value of “00010100” at position “001”.

According to the grayscale information of each pixel in F, it is converted into the corresponding quantum sequence in turn to realize the unique mapping process of grayscale information and position information. For example, the quantum circuit diagram of a pixel with the grayscale value 00010100 at position Y = "0", X = "01" is shown in Fig. 8.

Iterating through the 8-pixel information in F, the quantum sequence corresponding to all pixel information will be obtained, and the preparation of all grayscale information in F is completed.

(5) Measure the quantum image

The INCQI stores the grayscale information and position information in the classical image F in the superposition state of the quantum sequence, and by the measurement operation of each quantum bit, the quantum grayscale image system will collapse and finally output the position information and grayscale information of each pixel in the form of probability amplitude. In order to obtain the complete image information, it is necessary to make several measurements of the quantum preparation circuit. If the number of measurements is too small, the complete image information cannot be obtained; if the number of measurements is too large, the running time of the program will be affected. Therefore, it is necessary to choose the right number of measurements when running the program. In this paper, the different number of measurements are compared for the preparation of the INCQI quantum images of image F. The analysis is performed in terms of the time spent on the measurement, the number of measurement results, and the completeness of the state of the measurement results. Table 5 shows the experimental results of comparing 2 × 4 size INCQI quantum images with a different number of measurements.

**Table 5:** Comparison experimental results of 2 × 4 INCQI quantum image under different measurement times.

Number of measurement	Time/s	Number of measured results	Result integrity (Y/N)
8	3.2	6	N
16	3.3	7	N
32	3.6	8	Y
64	3.5	8	Y
128	3.3	8	Y
256	3.9	8	Y
512	3.7	8	Y
1024	4.7	8	Y
2048	5.2	8	Y
4096	5.9	8	Y
8192	9.5	8	Y

As can be seen from the table, when the number of measurements is less than 32, the number of results measured is less than 8. Therefore, when the number of measurements is too small, the image information obtained is incomplete. Besides, the program running time gradually increases as the number of measurements increases.

Since the maximum number of measurements in the IBMQ platform is limited to 8192, only a maximum of 8192 quantum image tests are performed in the experiments of this paper. Table 6 shows the output results after 1024 quantum image measurements.

**Table 6:** The output result after 1024 quantum image measurements.

No	Output results	No	Output results
1	00000000000	5	10000101000
2	00100011110	6	10101101001
3	01001001100	7	11000100110
4	01100010011	8	11111111111

**Figure 9:**
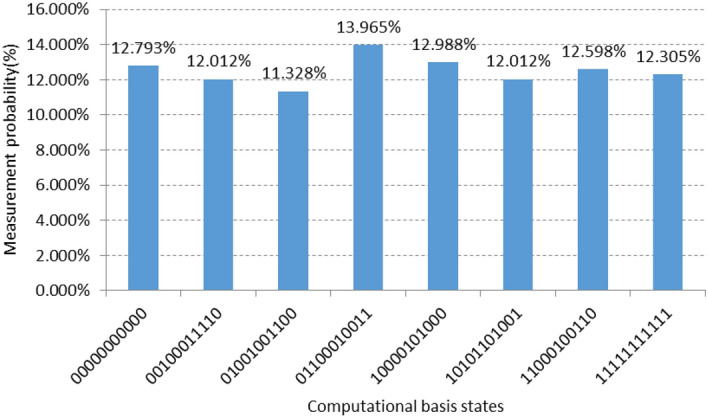
Probability histogram of different pixels obtained after 1024 quantum measurements.

Take the fourth output result "01100010011" as an example; this binary string from left to right corresponds to the output result of the quantum line from bottom to top. To facilitate observation, the binary string can be reversed before, and after the reversal, the output becomes "11001000110", at this time, the binary string from left to right and the quantum line from top to bottom, then the first 8 bits "11001000" is the grayscale information; the last 3 bits are the position information, where the position information in the y-direction is "1"; the position information in the x-direction is "10"; Fig. 9 shows the probability histogram of different pixels after 1024 quantum measurements.

As can be seen from the figure, the whole quantum image system stores 8 ground states simultaneously and collapses into the ground state after measurement, with different ground states having different probability amplitudes.

(6) Quantum sequence visualization

After the quantum image is measured and the classical information is obtained in terms of probabilistic amplitude, a subsequent operation is required for the whole quantum grayscale image system, i.e., the feedback probabilistic information is pictorialized and the image information is reduced for display. The quantum sequence information of the superposed state after the quantum operation is plotted in the corresponding size of the grayscale image. For example, to picture the quantum state of the 4th output result, first, the binary sequence "01100010011" is inverted to get "11001000110", and then the first 8 bits "11001000" are converted from binary sequence to decimal "200", the y-direction coordinates of the pixel are converted to "1", the x-direction coordinates are converted to "2", and finally the gray value of the pixel with coordinates "(1,2)" is set to "200" on a blank imageboard according to the above classical information, and so on, can complete the whole image of the assignment operation.

## Discussion

When performing quantum image preparation experiments on the IBMQ Quantum Cloud platform, it takes at least a few seconds to get the output results regardless of the number of measurements. This is caused by the waiting time and network latency for quantum program jobs to be submitted to the quantum computer backend queue. Quantum measurement experiments are performed in the IBMQ platform, and the actual quantum program runs in the backend in much less time than the measurement time during the experiment. In this paper, the feasibility of the quantum image control circuit for preparing INCQI quantum images is verified by designing a quantum circuit for the INCQI and implementing a 2 × 4 quantum image preparation using the IBMQ platform Qiskit software package. Without considering the memory space and time cost, quantum images of different sizes can be prepared by using basic quantum logic gates and referring to the design flow of the INCQI quantum image control circuit. In this paper, we only verify the quantum image preparation of the INCQI in 2 × 4 grayscale images, but not for large-size color images, mainly because of the limitation of the current IBMQ platform on the number of operable quantum bits. In addition, the time cost and memory space required to implement the simulation process of quantum algorithms using classical computers increases exponentially with the number of quantum bits. Therefore, preparing a large-size quantum image is almost unattainable at present. Quantum simulation experiments under classical computers are subject to many limitations and cannot accomplish the research of quantum algorithms in the real sense. At present, many quantum algorithms with low quantum bits have been sufficiently demonstrated, which provides a new direction of thinking for the subsequent development of quantum computing theory.

The INCQI proposed in this paper can well handle images with inconsistent horizontal and vertical position sizes and subsequent transformation operations of quantum images, and facilitate multi-channel quantum image transformation. Also, the development of programmable quantum computers and quantum programming languages is relatively cutting-edge, and there are only a few open-source quantum computers available for researchers in the world, therefore, there is a lack of literature on quantum image processing based on quantum programming languages. In the following research, we will focus on the combination of quantum image processing with quantum computers and quantum programming languages, not only on the theoretical analysis of quantum images but also on the implementation of quantum images on quantum computers.

## Conclusions

In this paper, we propose the INCQI based on the NCQI, which is capable of processing color images and using subsidiary quantum bits to facilitate multichannel quantum image transformation. Besides, the INCQI can realize the secondary acceleration of quantum image representation. Finally, the corresponding tests were conducted on the IBMQ platform to realize the conversion process from classical digital image information to quantum image information. In addition, a quantum circuit was designed using basic quantum logic gates, and the preparation process of INCQI quantum images on the IBMQ quantum computer was elaborated to verify the feasibility and effectiveness of the algorithm. The experiments show that the quantum image control circuit designed based on the INCQI can successfully prepare quantum image system, which verifies the feasibility of INCQI quantum image preparation process.
